# Preparation of Tea Tree Essential Oil–Chitosan Microcapsules and Its Effect on the Properties of Water-Based Coating

**DOI:** 10.3390/polym17070849

**Published:** 2025-03-22

**Authors:** Ye Zhu, Xiaoxing Yan

**Affiliations:** 1 Co-Innovation Center of Efficient Processing and Utilization of Forest Resources, Nanjing Forestry University, Nanjing 210037, China; zhuye@njfu.edu.cn; 2 College of Furnishings and Industrial Design, Nanjing Forestry University, Nanjing 210037, China

**Keywords:** tea tree essential oil, microcapsules, water-based, antibacterial coatings

## Abstract

The main chemical components of tea tree essential oil (TTO) are monoterpene compounds, including terpine-4-ol,1,8-cineole, para-cymene and γ-terpene. Among them, terpine-4-ol and 1,8-cineole are the main antibacterial components. The microcapsules were prepared by orthogonal experiments with the core–wall ratio, emulsifier concentration, mass ratio of Tween-80 to SDBS and oil–water ratio as variables. Through the analysis of the yield and coverage rate of nine kinds of microcapsules, the concentration of emulsifier was determined as the most influential factor for TTO–chitosan microcapsules, and six kinds of microcapsules were prepared by a single-factor experiment. With the increase in emulsifier concentration, the antibacterial rate of *Escherichia coli* increased first and then decreased, the antibacterial rate of *Staphylococcus aureus* increased first, decreased and then increased, and the antibacterial effect of *Staphylococcus aureus* was better than that of *Escherichia coli*. When the emulsifier concentration was 4% (13# microcapsule), the overall performance of the coating was better, the microcapsule dispersion was the most uniform, showing a round spheroid shape, and the particle size was mostly distributed between 4 and 8 μm. The antibacterial rate against *Escherichia coli* was 72.95%, and the antibacterial rate against *Staphylococcus aureus* was 75.81%. The color difference was 2.77, the glossiness at a 60° incidence angle was 22.8 GU, and the visible light transmittance was 87.80%. The roughness was 0.304 μm, and the elongation was 17.47%. The research results provide a technical reference for the application of an antibacterial water-based coating on a wood surface.

## 1. Introduction

The tea tree, also called Melaleuca alternifolia, belongs to the genus Melaleuca in the myrtle family [1]. Tea tree essential oil (TTO) is a colorless to light yellow oily liquid extracted from tea tree by steam distillation, with sharp camphor odor and menthol coolness [2]. TTO has a broad antibacterial spectrum and strong antibacterial activity, and bacteria cannot develop resistance to TTO. TTO is the most active natural antibacterial agent found so far [3,4]. The TTO has a bactericidal effect in the form of a membrane destroyer. The TTO acts by increasing the permeability and loss of film integrity of the liposome system that causes lysis, and ultimately leads to bacterial death by ion leakage and inhibited respiration [5,6,7]. The TTO is mainly composed of monoterpenes, sesquiterpenes, alkenes and alcohols. The main components of TTO are monoterpene compounds, including terpine-4-ol,1,8-cineole, para-cymene and γ-terpene [8]. Among them, 1,8-cineulin, 4-terpinene, γ-terpinene, α-terpinene and cymene are the main effective components to inhibit *Staphylococcus aureus* and *Escherichia coli*, and the antibacterial mechanisms of each component are different, playing a synergistic antibacterial role [9,10,11,12]. Chung et al. used optical biosensors to measure the antibacterial activity of essential oils (tea tree, chamomile and eucalyptus) on cells through their binding interactions and affinity. They found that the affinity of cell binding to tea tree was about twice that of other essential oils and was stronger than other essential oils [13].

Chitosan (CS) is a kind of straight chain natural polymer which is mainly glucosamine and a small amount of n-acetylglucosamine connected by β-1,4-glucoside bonds [14,15,16]. It is the only basic polysaccharide among natural polysaccharides [17]. Because CS contains free amino groups, it can be protonated by H^+^ to form a polycationic natural polymer, which has a natural spectral antibacterial property, biocompatibility and degradability [18,19]. As a natural, safe and non-toxic antibacterial preservative, CS has a wide range of application scenarios in the fields of clothing [20], food [21] and bio-medicine [22,23]. However, the antibacterial ability of CS itself is weak, so it is an urgent problem to improve the antibacterial ability and antibacterial persistence of CS. Due to its excellent slow-release protection and the ability to change the physical state, microcapsule technology can be used to coat antibacterial agents or antibacterial substances with poor antibacterial durability and insufficient antibacterial spectrum to make microcapsule antibacterial agents, which can improve the processing performance of antibacterial agents and expand their application range [24,25,26]. Among them, the antibacterial agent coated internally is called the core material, and the outer material is called the wall material. The physicochemical properties of the wall material are the key to the effect of antibacterial microcapsule coating [27,28]. CS molecules contain free amino groups, which are dissolved in solution to form salts, showing cationic properties, and then polymerized to form membranes in the presence of anions [29,30]. CS is an ideal wall material for preparing antibacterial microcapsules. Wjesirigunawardana et al. prepared lime oil (LO) microcapsules using CS and gum arabic as wall materials and LO as core material by composite coacervating method, and developed an anti-oxygen and antibacterial intelligent cotton fabric containing LO microcapsules. The antibacterial activity of four kinds of fine bacteria was evident before and after washing and cleaning [31]. Microcapsules prepared with CS and TTO can be synergistically antibacterial. Zhang et al. successfully prepared microcapsules with porous starch adsorbing TTO as the core material, sodium alginate and CS as the wall material, and glutaraldehyde as the crosslinking agent by polyelectrolyte complex condensation method, which proved the slow-release performance of microcapsules [32].

Using CS as the wall material and TTO as the core material, an orthogonal experiment and single-factor experiment were conducted to test and characterize the coating rate, yield, morphology and particle size of microcapsules, so as to explore the best process parameters for preparing microcapsules. The prepared microcapsules were added to the waterborne coatings with 5% content to prepare the paint film. The effects of different microcapsules on the macro-morphology, micro-morphology, optical properties, mechanical properties and antibacterial properties of the paint film were analyzed.

## 2. Materials and Methods

### 2.1. Materials and Equipment

The detailed list of raw materials and information required for this test was shown in Table 1, and the detailed list of equipment required was shown in Table 2. The coating preparation mold was made of silica gel with a size of 50 mm × 20 mm × 10 mm. The slide was made of glass with a size of 25.4 mm × 76.2 mm. The petri dish was 90 mm in diameter. The polyethylene film size was 40 mm × 30 mm × 0.08 mm. The paint was Dulux waterborne varnish [33]. *Escherichia coli* was the second-generation standard strain ATCC25922, and *Staphylococcus aureus* was the second-generation standard strain ACTT6538.

### 2.2. Microcapsule Preparation Method and Experimental Design

In this study, an orthogonal test and single-factor test were used to explore the optimal process parameters for the synthesis of microcapsules. Firstly, a four-factor and three-level orthogonal experiment was designed, as shown in Table 3 [34,35,36,37]. Nine different microcapsules were obtained by controlling the core–wall ratio of TTO to CS, emulsifier concentration, the mass ratio of Tween-80 to SDBS, and oil–water ratio during the preparation of the microcapsules. The specific preparation parameters are shown in Table 4. # was used to distinguish the serial number representing the prepared microcapsule from other numbers, such as 1# microcapsule. The results of the yield and coverage rate of these nine microcapsules were compared and analyzed to determine the primary and secondary levels of each factor, the optimal levels and the factors that have the greatest influence on the results.

Wall material preparation: A certain amount of acetic acid was weighed in deionized water to prepare 1.0% acetic acid solution, and 1.000 g chitosan powder was added. A magnetic stirrer was added into the beaker, the temperature of the water bath was set at 45 °C, the rotating speed was 600 rpm, and the reaction time was 1 h to obtain the wall material chitosan solution [38].

Core material emulsification: The emulsifier SDBS and Tween-80 were mixed into deionized water proportionally and TTO was added into the beaker. The temperature of the water bath was set to 45 °C and the rotating speed to 1200 rpm. After a 1 h emulsification, the liquid was ultrasonicated for 10 min, and then emulsified for 30 min to obtain a uniformly dispersed core material solution [39].

Microencapsulation: The core material emulsion was placed in the magnetic stirrer at 45 °C and 600 rpm. The wall material CS solution was absorbed with an eyedropper and added to the core material emulsion drop by drop. Acetic acid was added to adjust the pH to about 4. The emulsion was microencapsulated for 1 h. The 0.400 g of sodium tripolyphosphate (STPP) was weighed to 19.600 g of deionized water. The STPP solution was dropped into TTO–CS solution, and was cross-linked for 3 h. Materials used in the orthogonal experiment are shown in Table 5.

Spray drying: The microencapsulated solution was left to stand for 12 h. The temperature of the spray dryer was set to 110 °C and the feed rate to 200 mL/h. TTO–CS microcapsules were obtained by collecting the powder in the spray dryer [40].

Through the analysis of the coverage rate and yield of nine kinds of microcapsules, it was found that the emulsifier concentration was the most influential factor in the preparation of TTO–CS microcapsules. A single-factor experiment was set up with gradients of 1.0%, 2.0%, 3.0%, 4.0%, 5.0% and 6.0% to further optimize the specific preparation parameters of microcapsules. The test materials are shown in Table 6.

### 2.3. Coating Preparation Method

The prepared single-factor microcapsules were added to the water-based topcoat with 5% mass fraction and mixed evenly to obtain 1.0 g of the topcoat. The obtained top coating was evenly coated in the silicone mold and on the glass plate, and dried flat at room temperature for 20 min. The mold was transferred to the oven with a set temperature of 55 °C for 30 min, and then removed after the quality of the coating was constant.

### 2.4. Test and Characterization

#### 2.4.1. Yield and Coverage Rate Testing

Yield: The total mass of all raw materials was recorded as *M*_1_, and the mass of the obtained microcapsules after drying was recorded as *M*_2_. The formula for calculating the yield was as follows:

(1) $$Yield=\frac{M_{2}}{M_{1}}\times 100\%\;$$

Coverage rate: The microcapsule powder was weighed with the mass of *M*_3_, and it was ground fully with a mortar to destroy the wall material of the microcapsule. The ground powder was put into glassware, ethanol was added to the powder and soaked for 48 h. The soaked product was rinsed with ethanol and filtered. The filter paper and wet blank were put into the oven at 60 °C, and dried until the mass was constant and the weight was unchanged. The resulting material was the residual wall material. The total mass of wall material after drying was *M*_4_. The coverage rate was calculated as in Equation (2).

(2) $$Coverage\;rate=\frac{M_{3}-M_{4}}{M_{3}}\times 100\%\;$$

#### 2.4.2. Micromorphology

The microcapsules were observed by Zeiss optical microscope (OM), and the microcapsules and the prepared film were analyzed by scanning electron microscope (SEM). The prepared microcapsule and coatings were, respectively, pasted on the sample table; the test sample was placed in a specific position after gold spraying treatment, and the observation multiple and focal length were adjusted for observation.

#### 2.4.3. Chemical Composition Analysis

The chemical compositions of the core material, wall material, microcapsule and the prepared antibacterial coatings were analyzed by Fourier infrared spectroscopy (FTIR). According to ISO 20579-3:2021 [41], the powder sample should be made into thin slices by using the tablet pressing mechanism, and then tested and characterized.

#### 2.4.4. Optical Performance Test

Color difference: According to GB/T 11186.3-1989 [42], the color difference of the coating was tested using a portable color difference meter. The color difference meter was calibrated for testing. A place on the coating was randomly measured, and *L*, *a* and *b* were obtained by averaging three measurements. The test values of coating without microcapsules were *L*_1_, *a*_1_ and *b*_1_, and the test values of coating with microcapsules were *L*_2_, *a*_2_ and *b*_2_. *ΔL* represents the brightness difference of the coating; *Δa* represents the difference between the red and green coating; *Δb* represents the difference between the yellow and blue coating. The color difference *ΔE* was calculated according to Formula (3), where *ΔL* = *L*_2_ − *L*_1_, *Δa* = *a*_2_ − *a*_1_ and *Δb* = *b*_2_ − *b*_1_.

(3) $$\Delta E={[({\Delta L)}^{2}+({\Delta a)}^{2}+\left({\Delta b)}^{2}\right]}^{\frac{1}{2}}$$

Glossiness: According to GB/T 4893.6-2013 [43], the glossiness of the coating was tested. The glossiness was measured at the incidence angles of 20°, 60° and 85°. According to Formula (4), the loss of light of the coating was calculated. *G_L_* was the loss of light, *G*_0_ was the gloss of the coating without microcapsules and *G*_1_ was the gloss of the coating with microcapsules.

(4) $$G_{L}=\frac{G_{0}-G_{1}}{G_{0}}\times 100\%$$

Transmittance: According to ISO 2813:2014 [44], an ultraviolet spectrophotometer was used to test the transmittance of the coating, and the wavelength range of the test was 380 nm–780 nm in the visible band.

#### 2.4.5. Roughness and Tensile Testing

Roughness: According to ISO 25178-601:2025 [45], a roughness tester was used to test and the roughness value was recorded. The coated glass plate was placed on the testing table, the position of the stylus was adjusted to contact the coating and the roughness of the coating was tested and recorded.

Elongation: According to ASTM D2370-16 (2021) [46], the coating prepared by the silica gel mold was demolded, and the tensile test was carried out by a universal mechanical testing machine. The elongation of the coating at the breaking point was calculated by Formula (5), where *e* was elongation, *L*_0_ was the original length of the sample and *L* was the length at breaking. The tensile strength of the coating was calculated by Formula (6) and the tensile model was calculated by Formula (7). *P* is the maximum load (N), *b* is the sample width (mm), *d* is the sample thickness (mm) and *σ* is the stress (MPa). The *e* is strain, that is, elongation. *E* is the elastic modulus (GPa).

(5) $$e=\frac{L-L_{0}}{L_{0}}\times 100\%$$

(6) $$\sigma =\frac{P}{b\times d}\;$$

(7) $$E=\frac{\sigma }{e}$$

#### 2.4.6. Antibacterial Property

*Escherichia coli* and *Staphylococcus aureus* were selected for the test operation according to the antimicrobial determination method specified in GB/T 21866-2008 [47]. Firstly, live bacteria were cultured. A total of 24 g of agar medium was weighed to prepare agar plate medium with 1000 mL distilled water and sterilized at 121 °C for 1 h. The bacteria on the inclined medium were transferred to the flat nutrient agar medium using the sterilized inoculation ring. The temperature of the constant temperature and humidity chamber was set at 37 °C and the humidity was 98%. The agar medium was cultured for 18–20 h. The culture preserved on the inclined side was the fresh culture preserved for less than one month. A total of 9 g of nutrient broth and 500 mL of distilled water was weighed to prepare the bacterial suspension, which was sterilized at 121 °C for 1 h. The inoculating ring was used to scrape 1–2 rings of bacteria from the live bacteria on the agar medium and the bacteria were added to the broth culture medium. The broth culture medium was diluted to the test bacterial suspension with a concentration of 10^6^ cfu/mL for use. The polyethylene film was cut and sterilized for later use. The 0.1 mL bacterial suspension drops were taken with a pipette gun and placed on the glass plate. The sterilized tweezers were used to cover the surface of the test piece with polyethylene film to ensure that the bacterial suspension was dispersed on the surface of the test piece without bubbles. The glass plate was placed in a disposable culture dish and cultured at constant temperature and humidity for 24 h.

A certain amount of sodium chloride was added to distilled water and dissolved by heating to prepare 0.85% eluent. The 20 mL of eluent was added and the sample paint film and cover film were rinsed repeatedly. After mixing the eluent evenly, the 0.5 mL was inoculated in flat nutrient agar medium and cultured in a constant temperature and humidity chamber at 37 °C and 98.0% for 48 h. According to GB/T 4789.2-2022 [48], a colony counter was used to measure and record the number of colonies in the medium, and the number of colonies multiplied by 1000 was the actual recovered colony value of each sample after 48 h of culture. The calculation Formula (8) of antibacterial rate was as follows: where *R* represented antibacterial rate and the unit was %; *B* represented the average number of recovered colonies of pure film samples after 48 h. *C* represented the average number of bacteria recovered from the antibacterial coating sample after 48 h, and the unit was CFU/piece.

(8) $$R=\frac{B-C}{B}\times 100\%$$

## 3. Results and Discussion

### 3.1. Yield and Coverage Rate

The preparation of TTO–CS microcapsules with high yield with less raw materials can save resources and improve efficiency, which has important significance for its production and practical application. Table 7 shows the analysis of yield results obtained by the orthogonal experiment. Among the nine groups of microcapsule samples, sample 9# had the highest output of 3.20 g, followed by sample 6# with 3.12 g. Sample 6# had the highest yield of 42.92%, followed by sample 9#, which was 40.51%. By comparing the size of the mean data, it can be concluded that the optimal level was A2 B3 C2 D2: the core–wall ratio was 1.2:1, the emulsifier concentration was 4%, the mass ratio of Tween-80 to SDBS was 1:3, and the oil–water ratio was 3:2. According to the comparison of the range results, the main and secondary levels of the influence of each factor on the microcapsule yield were B > C > A > D, and the most influential factor B was the concentration of the emulsifier, followed by the mass ratio of Tween-80 to SDBS, followed by the core–wall ratio, and finally, the oil–water ratio. Table 8 presents the analysis of variance results for the yield. The variance results for the four factors were the same as the range results, and the four influencing factors were not significant.

Coverage rate refers to the ratio of core TTO to the overall quality of microcapsules, which is an important factor affecting the antibacterial performance of TTO–CS microcapsules, and is one of the important bases for measuring the preparation results of microcapsules. Theoretically, the higher the coverage rate, the higher the core material content and the better the antibacterial effect [49]. Table 9 shows the analysis of the coverage rate results obtained by orthogonal experiments. The coverage rate of sample 4# was the highest (67.5%), followed by sample 1# (62.5%). By comparing the mean data size, the optimal level was obtained as A1 B1C3 D3: the core–wall ratio was 1:1, the emulsifier concentration was 2%, the mass ratio of Tween-80 to SDBS was 1:4, and the oil–water ratio was 2:1. According to the comparison of the range results, the main and secondary levels of influence of each factoring into the microcapsule coverage rate were B > A > C > D, and the most influential factor B was the emulsifier concentration, followed by the core–wall ratio, followed by the mass ratio of Tween-80 to SDBS, and finally, the oil–water ratio. Table 10 presents the analysis of the variance results of the coverage rate. The variance results of the four factors were the same as the range results, and the four influencing factors were not significant.

The single-factor experiment was designed by combining the optimum preparation process parameters of yield and coverage rate from the orthogonal experiment. The concentration of the emulsifier with greater comprehensive influence was set as a variable, the core–wall ratio, the mass ratio of Tween-80 to SDBS and the oil–water ratio were fixed factors. The increase in core material content meant the increase in antibacterial agent content, and the antibacterial rate was improved to a certain extent. When the core–wall ratio was small, the wall material was excessive and the wall was thickened, so the core–wall ratio was set to 1.2:1. A higher coating rate represented a higher content of core material in microcapsules, which increased the release of core material content and improved the antibacterial property of microcapsules. Therefore, the results of microcapsule coverage rate were referred to in the single-factor test. After considering the above factors, the remaining three preparation conditions were fixed: the core–wall ratio was 1.2:1, the mass ratio of Tween-80 to SDBS was 1:4 and the oil–water ratio was 2:1.

Table 11 shows the yield and coverage rate results for six kinds of microcapsules in the single-factor test. The highest yield of sample 14# was 35.19%, and the highest coverage rate of sample 13# was 57.5%. With the concentration of the emulsifier gradually increasing, the production first increased and then decreased, and the coverage rate showed a trend of first increasing, then decreasing and finally increasing. The addition of emulsifier is not a case of the more, the better, and the addition of emulsifier in a certain range can help maintain the balance of water and oil and improve the stability of the emulsion [50]. The amount of surfactant required to form a stable emulsion between the wall and the core was certain. When this amount was exceeded, the effect of the emulsifier was not obvious, and even affected the embedding stability. Studies have shown that too much emulsifier caused the core material to disperse too small, increased the surface area and could not be effectively coated.

### 3.2. The Micromorphology of Microcapsules

**Figure 1:**
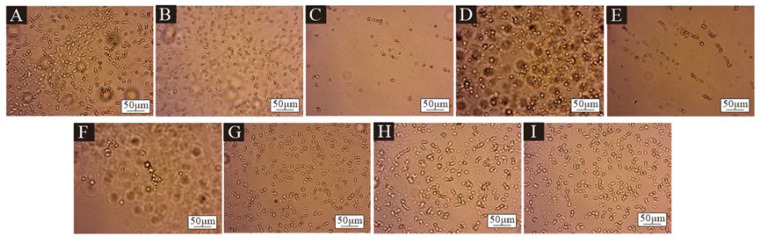
Microscopic diagram of microcapsule samples in the orthogonal test: (**A**) 1#, (**B**) 2#, (**C**) 3#, (**D**) 4#, (**E**) 5#, (**F**) 6#, (**G**) 7#, (**H**) 8# and (**I**) 9#.

**Figure 2:**
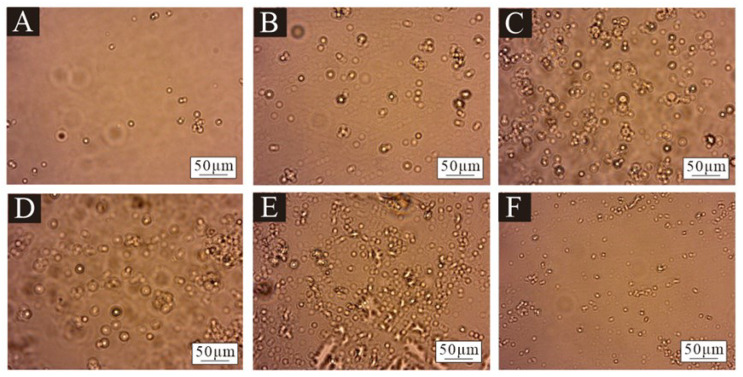
Microscopic diagram of microcapsules samples in the single-factor test: (**A**) 10#, (**B**) 11#, (**C**) 12#, (**D**) 13#, (**E**) 14# and (**F**) 15#.

Figure 1 presents the microcapsule microscope image obtained by the orthogonal test, in which A–I corresponds to microcapsule samples 1#–9#. Because the core material and the wall material had different absorption and reflection effects on light, the shell–core structure of the microcapsule could be seen. The inner bright spot of the core material represented the core material and the outer black circle represented the wall material. It was preliminarily proved that there were two kinds of materials, wall material and core material, and the core material was covered by the wall material. Among them, 7# and 9# samples had the best morphology, more microcapsules, uniform dispersion, less aggregation, an approximately circular shape and an obvious shell core structure. The morphology of 3# and 6# samples was poor, with fewer microcapsules and obvious agglomeration. Figure 2 shows the microcapsule microscopy under the single-factor test. The six microcapsules were uniformly dispersed without obvious agglomeration. However, there were fewer microcapsules in the 10# sample, and the emulsifier for preparing microcapsules was 1.0%. This indicated that the emulsifier content was small and the emulsification was not complete, which was not conducive to the formation of microcapsules.

**Figure 3:**
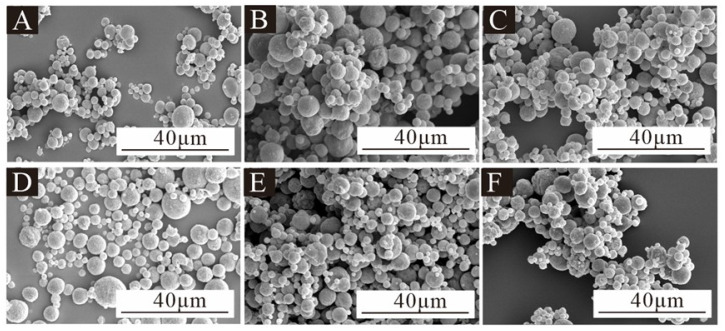
SEM images of microcapsules in the single-factor test at low magnification: (**A**) 10#, (**B**) 11#, (**C**) 12#, (**D**) 13#, (**E**) 14# and (**F**) 15#.

**Figure 4:**
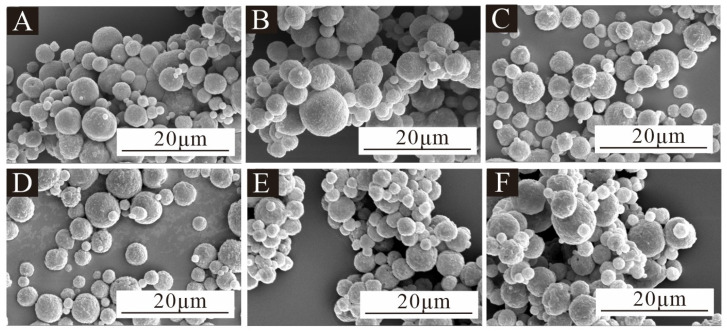
SEM images of microcapsules in the single-factor test at high magnification: (**A**) 10#, (**B**) 11#, (**C**) 12#, (**D**) 13#, (**E**) 14# and (**F**) 15#.

**Figure 5:**
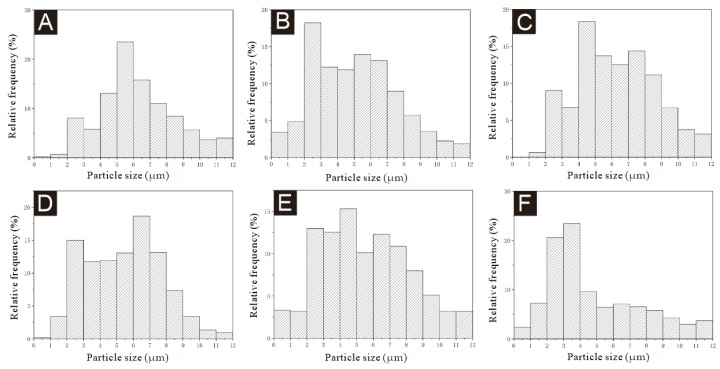
Particle size distribution of microcapsules samples in the single-factor test: (**A**) 10#, (**B**) 11#, (**C**) 12#, (**D**) 13#, (**E**) 14# and (**F**) 15#.

Figure 3 is the SEM image of microcapsules under low magnification for the single-factor test, Figure 4 is the SEM image of microcapsules under high magnification, and Figure 5 is the particle size distribution of the microcapsules. All six microcapsules were spherical. The surface of the microcapsules with 1.0% emulsifier concentration was smooth and there was a small agglomeration phenomenon. The particle size was mostly distributed in the 2–8 μm range, and the microcapsules with a particle size of 6–7 μm were the most frequent. The microcapsules with a 2.0% emulsifier concentration were spherical, round and full, and the particle size was agglomerated. The particle size was mostly distributed between 2 and 7 μm, and the microcapsules with a particle size of 2–3 μm were the most common. The microcapsules with a 3.0% emulsifier concentration dispersed more evenly, with a small amount of irregularly shaped substances, but most of them were rounded spheres, whose particle size was mostly distributed between 2 and 9 μm, and the microcapsules with a particle size of 4–5 μm were the most common. The microcapsules with a 4.0% emulsifier concentration had the most uniform dispersion, but the particle size varied greatly; most of them were distributed between 4 and 8 μm, and the microcapsules with a particle size of 5–6 μm were the most common. The microcapsules with a 5.0% emulsifier concentration were spheroid with a smooth surface and agglomeration. The particle size distribution was relatively uniform, mainly between 3 and 8 μm, and the particle size distribution was relatively concentrated, and the microcapsules with the particle size of 4–5 μm were the most common. The microcapsules with an emulsifier concentration of 6.0% were spherically shaped with a small amount of agglomeration. The particle size varied greatly, mainly between 2 and 4 μm. In general, the particle size distribution of 13# and 14# microcapsules was more uniform, and the microcapsule morphology was better. In the microencapsulation process, the microcapsule particle size and its distribution mainly depend on the initial core particle size. If the core material is liquid, the particle size of the microcapsule depends on the formation of the initial emulsion, and the emulsification effect of the core material directly affects the particle size and distribution of the microcapsule. In the emulsification process of the core material, the key factor affecting the particle size and distribution of liquid droplets is the uniformity of the liquid mixture by mechanical mixing, which depends on the structural design of the mixer, the mixing speed and the emulsification time. At the same homogenization speed, the longer the homogenization time, the better the emulsification effect. Under the same homogenization time, the higher the homogenization speed, the better the emulsification effect, the smaller and more uniform the emulsion droplets, the smaller the average particle size and the narrower the distribution of the microcapsules. The emulsification time and stirring speed of the six microcapsules in this experiment were all the same, so the particle size of the microcapsules was roughly the same.

### 3.3. Chemical Composition Analysis of Microcapsules

**Figure 6:**
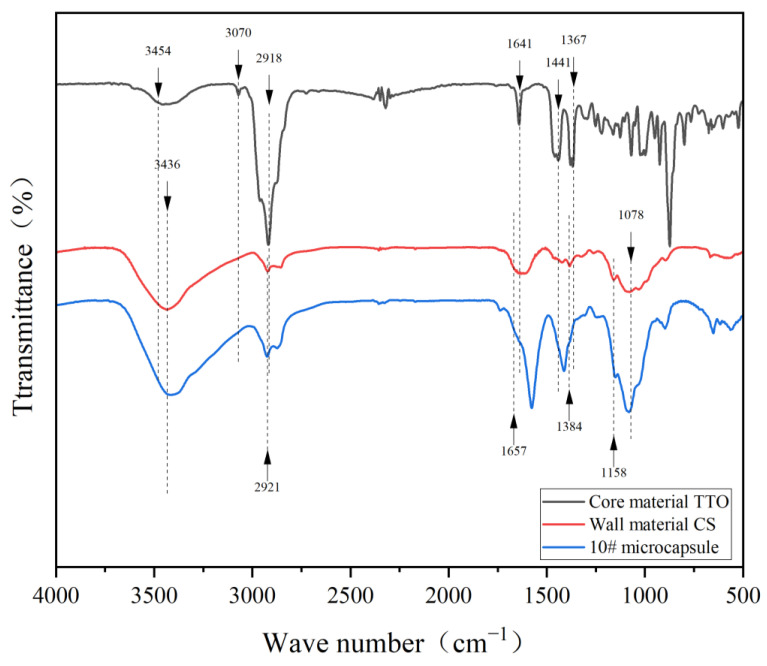
Infrared spectra of core material, wall material and 10# microcapsule.

The infrared spectra of core materials, wall materials and 10# microcapsules are shown in Figure 6. In the infrared spectrum of the CS, the absorption peak of -OH stretching vibration was at 3436 cm^−1^, that of saturated hydrocarbon -CH_2_ stretching vibration was at 2921 cm^−1^, that of C-O stretching vibration was at 1078 cm^−1^ and that of amide group I was at 1657 cm^−1^. The N-H group at 1384 cm^−1^ was the bending vibration of an amide, and C-O-C was the stretching vibration at 1158 cm^−1^ [51,52]. The main components of TTO are terpine-4-ol, α-terpinene, 1,8-cineole and other alkenes and alcohols. In the infrared spectrum of TTO, the absorption peak at 3070–3454 cm^−1^ was the stretching vibration of the hydroxyl group and the symmetric and asymmetric stretching vibration of the N-H bond in the amino group [53]. There was a strong unsaturated C-H vibration absorption peak at 2918 cm^−1^, an unsaturated C=C vibration absorption peak at 1641 cm^−1^ and a characteristic absorption peak at 1367–1441 cm^−1^ was the interaction of alcohol C-O and O-H in TTO [54,55]. In the absorption curve of microcapsules, absorption peaks of CS and TTO also appeared. Chemical components of CS and TTO were present in microcapsules, which proved that microcapsules were successfully coated.

### 3.4. Morphology and Chemical Composition Analysis of Water-Based Coating

**Figure 7:**
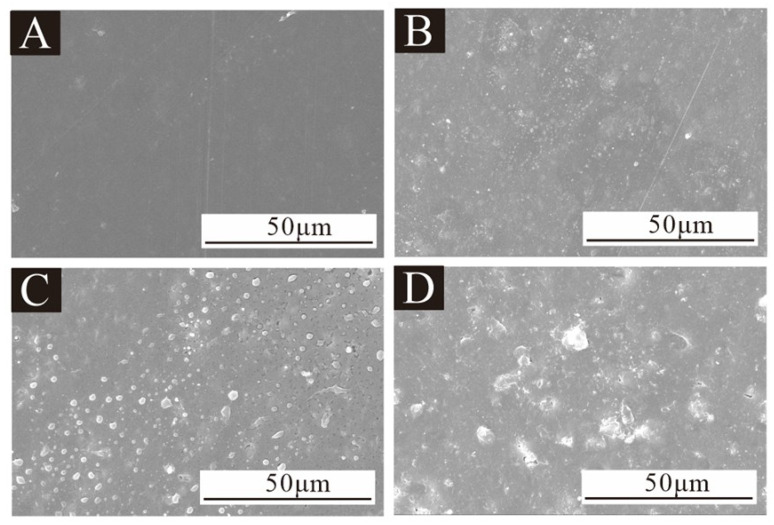
SEM images of coatings prepared by adding 5.0% microcapsules with different emulsifier concentration: (**A**) coatings without microcapsules and sample (**B**) 12#, (**C**) 13# and (**D**) 14# coatings.

Figure 7 shows the SEM images of the coating prepared by 12#, 13# and 14# microcapsules with 5.0% added content. When no microcapsules were added to the coating, the surface was relatively smooth. The surface of the coating with 5.0% microcapsules became rough and there were more protrusions. Among them, the coating surface of the 13# microcapsule was the roughest, and the coating surface of the 14# microcapsule was lower. There are more irregular aggregates in the 13# microcapsule, which makes the surface roughness of the prepared coating poor. The three kinds of microcapsule samples have the phenomenon of uneven dispersion and local adhesion, so the surface of the prepared coating is rougher than that of the pure coating.

**Figure 8:**
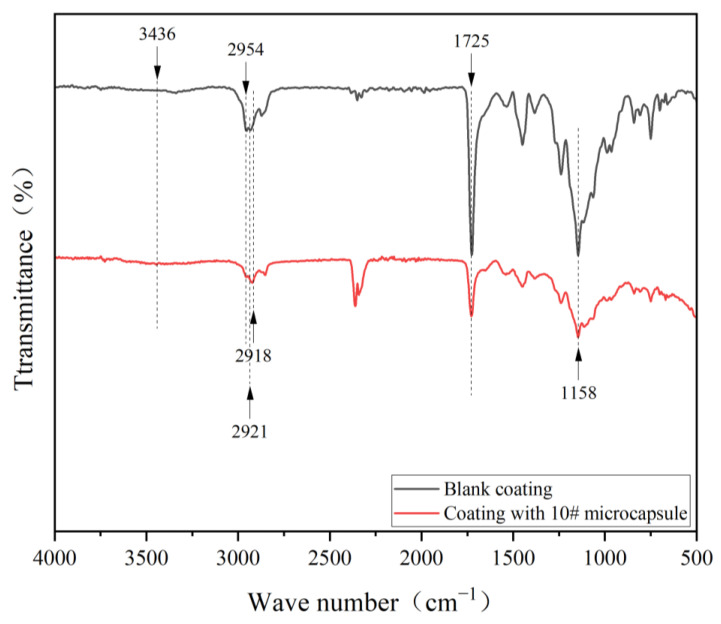
Infrared spectra of coating.

Figure 8 shows the infrared spectra of the coating with 10# microcapsules added and the blank coating. The O-H stretching vibration peak of both water-based coatings and core material TTO appeared at 3436 cm^−1^, the C-H bending vibration peak of water-based coatings was at 2954 cm^−1^, and the C=O stretching vibration peak of water-based coatings was at 1725 cm^−1^ [56]. The unsaturated C-H vibration absorption peak of TTO appeared at 2918 cm^−1^. The -CH_2_ stretching vibration peak of wall material CS appeared at 2921 cm^−1^, and the absorption peak of C-O-C was about 1158 cm^−1^. This proved that after the prepared microcapsule was added to the water-based topcoat, the wall material and core material components of the microcapsule still existed, and there was no chemical reaction between the microcapsule and the water-based topcoat.

### 3.5. Analysis of Antibacterial Properties of Coating

**Figure 9:**
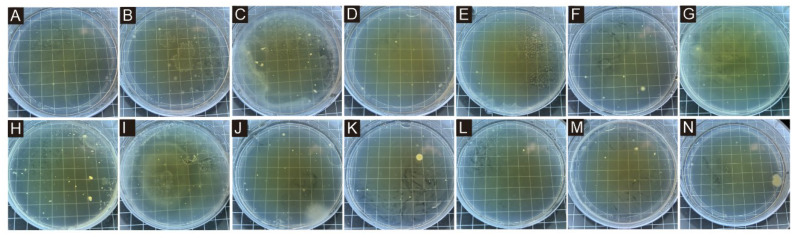
Colony recovery of coating from microcapsules with different emulsifier concentrations against *Escherichia coli*: (**A**) coating without microcapsules, (**B**) 10#, (**C**) 11#, (**D**) 12#, (**E**) 13#, (**F**) 14# and (**G**) 15# coating, and against *Staphylococcus aureus*: (**H**) coating without microcapsules, (**I**) 10#, (**J**) 11#, (**K**) 12#, (**L**) 13#, (**M**) 14# and (**N**) 15# coating.

**Figure 10:**
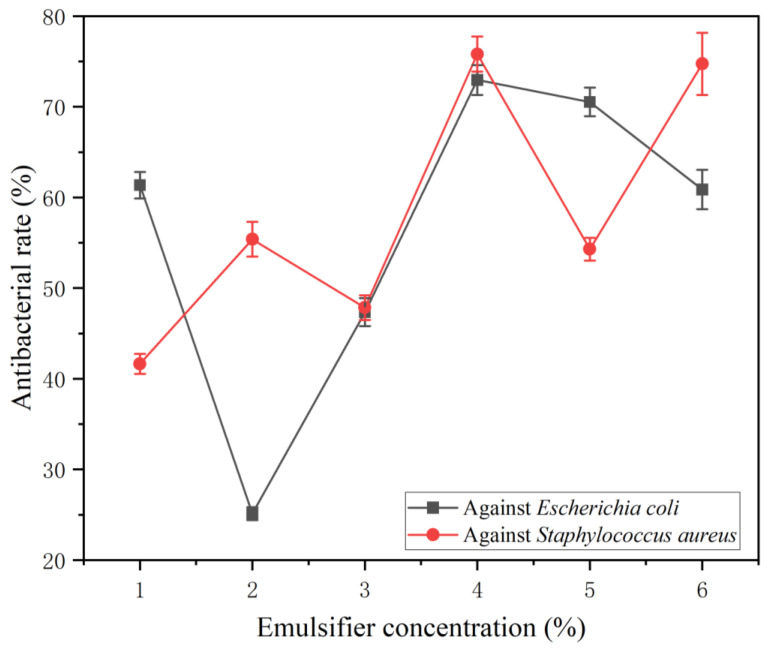
Antibacterial rate of coating of microcapsules with emulsifier concentration.

Table 12 shows the average recovered colonies and antibacterial rate of microcapsules with different emulsifier concentrations added. Compared with pure coating, the number of recovered bacteria in the coating with microcapsules was significantly reduced, which indicated that the addition of microcapsules improved the antibacterial property of the coating to a certain extent. Figure 9 shows the colony recovery diagram of the coating of different emulsifier concentration microcapsules, and Figure 10 shows the trend of the antibacterial rate of the coatings with different emulsifier concentration microcapsules against two kinds of bacteria. For *Escherichia coli*, with the increase in emulsifier concentration, the antibacterial rate of coating showed a trend of first decreasing, then increasing and finally decreasing. When the emulsifier concentration was 2.0%, that is, adding 11# microcapsules, the antibacterial rate of the coating was the lowest, which was 25.12%. When the emulsifier concentration was 4.0%, that is, adding 13# microcapsules, the antibacterial rate of the coating reached the maximum, 72.95%. The 13# microcapsule has the highest coverage rate and higher core material content, so it has a higher antibacterial rate. For *Staphylococcus aureus*, the antibacterial rate of the coating fluctuated with the increase in emulsifier. When the emulsifier concentration was 1.0%, that is, the 10# microcapsule was added, the antibacterial rate of the coating was the lowest (41.64%). Because the coverage rate of the 10# microcapsule is the lowest and the content of TTO is low, the antibacterial activity is poor. When the emulsifier concentration was 4.0%, that is, adding the 13# microcapsule, the antibacterial rate of the coating reached the maximum, 75.81%. When the concentration of the microencapsulated emulsifier was 4.0% (13# sample), the antibacterial rate of the prepared antibacterial coating against *Escherichia coli* and *Staphylococcus aureus* reached the maximum. The antibacterial rate of coating against *Staphylococcus aureus* was higher than that against *Escherichia coli*. TTO, a core material with antibacterial effect, can exert antibacterial effect on bacteria together with CS. Both the wall material and TTO with antibacterial effect effectively improve the antibacterial property of the coating.

### 3.6. Optical Properties Analysis of Coating

#### 3.6.1. Color Difference

The chroma value and color difference of the coating are shown in Table 13. Where *L* represents the brightness value of the coating, the light is positive and the dark is negative. The *a* represents the red–green value of the coating, with red being positive and green being negative. The *b* represents the yellow and blue value of the coating, with yellow being positive and blue being negative. From Table 13, with the increase in emulsifier concentration, the brightness value of the coating fluctuated little, the red–green value fluctuated up, the yellow–blue value fluctuated down, and the color difference of the coating fluctuated up. When the emulsifier concentration was 4%, that is, adding 13# microcapsules, the color difference of the coating was larger, which was 2.77. When the emulsifier concentration was 3.0%, that is, adding 12# microcapsules, the color difference of the coating was small, 0.70. The addition of six kinds of microcapsules had a low influence on the brightness of the coating, but a great influence on the color difference of the coating. Because the dispersion of microcapsules in water-based coatings is poor, there is an agglomeration phenomenon, which affects the flatness of the coating, so there will be a larger color difference.

#### 3.6.2. Glossiness and Light Loss Rate

Table 14 shows the changes of coating glossiness and light loss rate measured at three incident angles. Compared with the coating without microcapsules, the addition of microcapsules reduced the glossiness of the coating itself and increased the light loss rate. At the incidence angle of 20°, the difference between the glossiness of the coating changed little, and the concentration of emulsifier increased continuously; the gloss of the coating fluctuated and decreased. When the emulsifier concentration was 3.0%, that is, when the 12# microcapsule was added, the glossiness of the coating reached the maximum value of 15.7 GU, followed by 15.3 GU when the 10# microcapsule was added. At a 60° incidence angle, when the 10# microcapsule was added, the glossiness of the coating reached the maximum value of 27.9 GU, followed by the addition of the 12# microcapsule, which was 26.7 GU. At an 85° incidence angle, when the 12# microcapsule was added, the glossiness of the coating reached the maximum value of 33.3 GU, followed by the addition of the 10# microcapsule, which was 32.8 GU. At the incidence angle of 20°, the light loss rate of the coating was the smallest at 3.09 (adding 12# microcapsule), followed by 5.56 (adding the 10# microcapsule). At a 60° incidence angle, the light loss rate of the coating was the smallest at 4.78 (adding the 10# microcapsule), followed by 8.87 (adding the 12# microcapsule). At an 85° incidence angle, the light loss rate of the coating was the smallest at 10.24 (adding the 12# microcapsule), followed by 15.90 (adding the 10# microcapsule). Because the addition of microcapsules affects the flatness of the coating after curing, the surface of the coating becomes uneven, reducing the ability to reflect light, resulting in a decrease in glossiness and an increase in light loss rate [57]. When the emulsifier concentration was 1.0% and 3.0%, the glossiness and light loss rate of the coating were better.

#### 3.6.3. Transmittance

**Figure 11:**
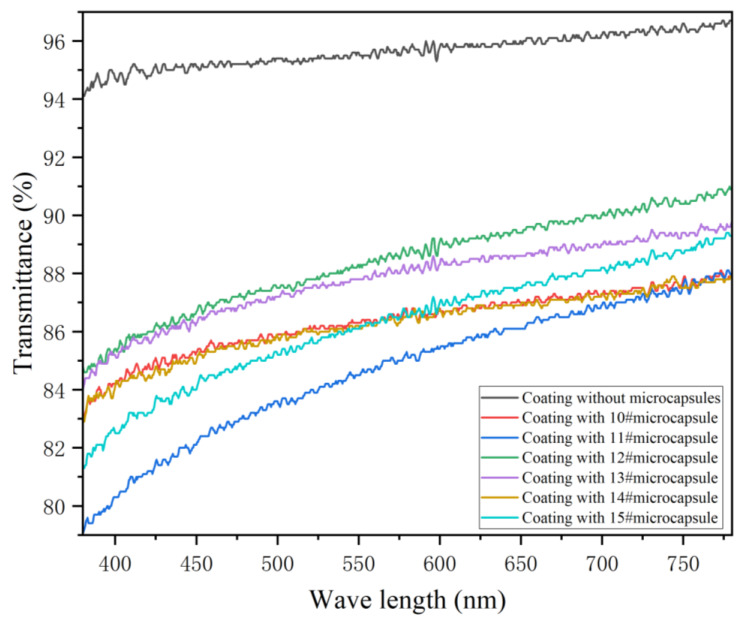
Light transmission curves of coatings with different emulsifier concentrations.

Table 15 and Figure 11 show the transmittance of the coating prepared by microcapsules with different emulsifier concentrations. The visible light transmittance of the coating containing microcapsules was lower than that without microcapsules. With the increase in emulsifier concentration, the transmittance of the prepared coating fluctuated in the visible light band. When the emulsifier concentration was 3.0%, that is, adding the 12# microcapsule, the transmittance of the coating was the highest, 88.42%, followed by the emulsifier concentration at 4.0%, that is, adding the 13# sample, for which the transmittance of the coating was 87.80%. When the concentration of emulsifier was 2.0%, that is, adding the 11# microcapsule, the transmittance of the coating was poor, at 84.67%. Because the microcapsules are added to the water-based topcoat, there is an agglomeration phenomenon, and the microcapsules are white opaque powder, which leads to the enhancement of the surface roughness of the prepared coating [58]. This reduces the transmission and reflection of the incident light, enhances the scattering phenomenon of light and thus reduces the transmittance of the coating. However, the coating as a whole was in a relatively transparent state, which had little impact on actual use.

### 3.7. Mechanical Properties Analysis of Coating

#### 3.7.1. Roughness

Table 16 shows the influence of microcapsules prepared with different emulsifier concentrations on the roughness of the coating. Compared with the coating without microcapsules, the addition of microcapsules increased the roughness of the coating. The roughness of the coating without microcapsules was less than 0.267 μm, and with the increase in emulsifier concentration, the roughness of the coating showed a fluctuating trend. When the emulsifier concentration was 4.0%, that is, adding the 12# microcapsule, the maximum roughness of the coating was 0.423 μm, followed by that of the emulsifier concentration of 6.0% (15# microcapsule), which was 0.344 μm. When the emulsifier concentration was 2.0%, that is, adding the 11# microcapsule, the roughness of the coating reached the minimum value of 0.269 μm. This is because the agglomeration phenomenon of sample 12# and 15# microcapsules is more serious, and reduces the dispersion of microcapsules in water-based coatings and increases the coating roughness [59].

#### 3.7.2. Elongation

**Figure 12:**
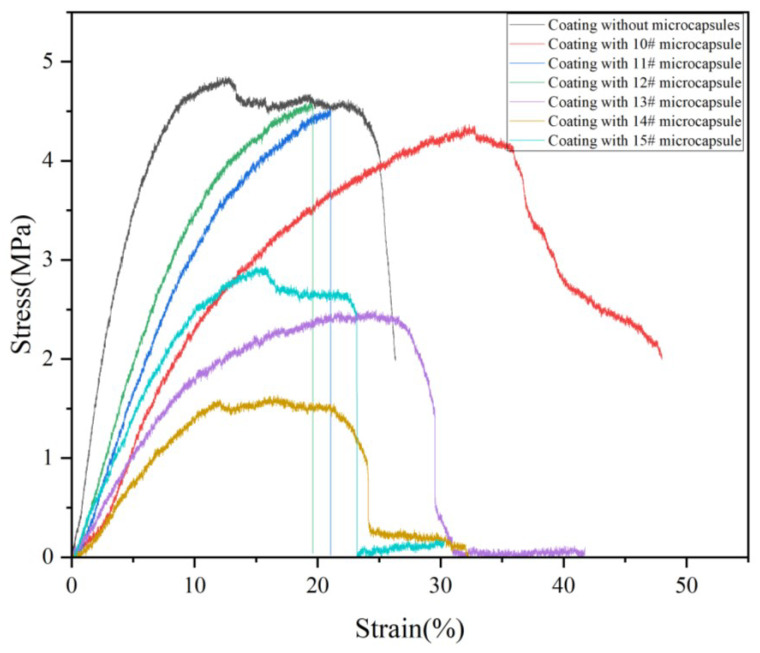
The influence of emulsifier concentration on coating tensile resistance.

Table 17 shows the effect of microcapsules prepared with different emulsifier concentrations on the coating elongation. The elongation is an important criterion to judge the ductility and toughness of the coating. The elongation of the coating without microcapsules was 20.73%. The elongation of the coating increased first and then decreased after microcapsules were added. This is because microcapsules are dispersed in the coating in a solid state, and the addition of microcapsules reduces the viscosity and elasticity of the paint itself [60,61,62]. Therefore, when subjected to tension, the relative tensile quantity of the coating becomes smaller and the elongation is reduced [63,64]. When the emulsifier concentration was 4.0%, that is, when the 13# microcapsule was added, the elongation of the coating reached the maximum, which was 17.47%. When the emulsifier concentration was 6%, that is, the 15# microcapsule was added, the elongation of the coating reached the minimum value of 4.8%. The influence of the six prepared microcapsules on the tensile resistance of the coating is shown in Figure 12. The coatings prepared by 10# and 13# microcapsules had a certain elastic region. This is because the microcapsule particles are distributed in the coating, which increases the density of the coating, thereby reducing the ductility of the water-based coating itself [65,66,67]. The stress of the coating without microcapsules was 4.84 MPa, and the tensile strength of the coating with microcapsules was reduced. It is because the addition of microcapsules makes the brittleness of the coating higher [68,69]. Among them, adding the 11# and 12# microcapsule coatings resulted in higher stress and better resistance to external force. The elastic modulus of the coating without microcapsules was 0.23 GPa; the elastic modulus of the coating with microcapsules was improved [70]. 

## 4. Conclusions

The orthogonal test and single-factor test were used to explore the optimum preparation technology of CS-coated TTO microcapsules, and the yield, coverage rate, morphology, particle size and chemical composition of the microcapsules were tested and analyzed. The results showed that the concentration of emulsifier was the most important factor for the preparation of microcapsules, and the optimum preparation process was as follows: the core–wall ratio was 1.2:1, the mass ratio of Tween-80 to SDBS was 1:4 and the oil–water ratio was 2:1. Six kinds of microcapsule samples prepared by the single-factor test were added into water-based coatings at a 5.0% additive content to prepare the coatings, and the morphology, chemical composition, antibacterial properties, optical properties and mechanical properties of the coatings were tested and explored. With the increase in emulsifier concentration, the antibacterial rate of the coating against *Escherichia coli* decreased first, then increased and finally decreased, and the antibacterial rate against *Staphylococcus aureus* increased in a fluctuating manner. The antibacterial rate of the coating against *Staphylococcus aureus* was higher than that against *Escherichia coli*. When the concentration of the microcapsule emulsifier was 4% (13# sample), the antibacterial rate of the prepared antibacterial coating against *Escherichia coli* and *Staphylococcus aureus* reached the maximum, 72.95% and 75.81%, respectively. With the increase in emulsifier concentration, the color difference of the coating fluctuated, the transmittance of the coating fluctuated in the visible band, the roughness of coating fluctuated and the elongation of the coating increased first and then decreased. In this paper, the TTO–CS microcapsule was prepared by a spray drying method to make water-based coatings with antibacterial properties, and a technical reference for the application of antibacterial microcapsules in water-based coatings was provided.

## Figures and Tables

**Table 1:** List of experimental materials.

Test Material	Purity	Producer
TTO	-	Jiangxi Zhonghuan Biotechnology Co., Ltd., Ji’an, China
CS	AR	Qingdao Boyite Biomaterials Co., Ltd., Qingdao, China
SDBS	AR	Shandong Longhui Chemical Co., Ltd., Jinan, China
Tween-80	AR	Shanghai Kanglang Biotechnology Co., Ltd., Shanghai, China
Acetic acid	AR	Henan Mingao Chemical Co., Ltd., Anyang, China
Sodium tripolyphosphate (STPP)	AR	Yuntianhua Group Co., Ltd., Kunming, China
Dulux primer	-	Dulux Paint Co., Ltd., Xiamen, China
Nutrient agar medium	-	Sinopharm Group Chemical reagent Co., Ltd., Shanghai, China
Nutrient broth medium	-	Sinopharm Group Chemical reagent Co., Ltd., Shanghai, China
Sodium chloride	AR	Sinopharm Group Chemical reagent Co., Ltd., Shanghai, China
*Escherichia coli*	-	Beijing Baozang Biotechnology Co., Ltd., Beijing, China
*Staphylococcus aureus*	-	Beijing Baozang Biotechnology Co., Ltd., Beijing, China

**Table 2:** Test equipment.

Equipment	Model	Manufacturer
High-precision balance	BSA323S	Sartorius Scientific Instruments (Beijing) Co., Ltd., Beijing, China
Heat-collecting magnetic stirrer	DF-101S	Shenzhen Dingxin Yi experimental equipment Co., Ltd., Shenzhen, China
Spray dryer	JA-PWGZ100	Shenyang Jingao Instrument Technology Co., Ltd., Shenyang, China
Blast drying oven	DHG-9423A	Shanghai Jinghong Experimental Equipment Co., Ltd., Shanghai, China
Scanning electron microscope	Quanta-200	Thermo Fisher Technologies, Waltham, MA, USA
Fourier infrared spectrometer	VERTEX 80 V	Bruker GMBH, Billerica, Massachusetts, Germany
Constant temperature and humidity box	THA150	Ellison Instrument Equipment (Shanghai) Co., Ltd., Shanghai, China
High-precision gloss meter	YG268	Shenzhen three Enshi technology Co., Ltd., Shenzhen, China
High-precision spectrophotometer	DC-23D	Color Spectrum Technology (Zhejiang) Co., Ltd., Hangzhou, China
Ultraviolet spectrophotometer	U-3900	Hitachi Scientific Instruments (Beijing) Co., Ltd., Beijing, China
Universal mechanical testing machine	AGS-X	Shimadzu Production House, Kyoto, Japan
Roughness meter	J8-4C	Shanghai Taiming Optical Instrument Co., Ltd., Shanghai, China
Ultrasonic emulsifier	XU-JY92-IIN	Shanghai Siniu Leiper Instrument Co., Ltd., Shanghai, China
Circulating water vacuum pump	SHZ-D (II)	Zhengzhou Huachen Instrument Co., Ltd.; Zhengzhou, China
Colony counter	XK97-A	Guangjurong experimental equipment business department, Chuzhou, China

**Table 3:** Factors and levels of the orthogonal experiment.

Levels	Factor A Core–Wall Ratio	Factor B Emulsifier Concentration (%)	Factor C Mass Ratio of Tween-80 to SDBS	Factor D Oil–Water Ratio
1	1:1	2.0	1:2	1:1
2	1.2:1	3.0	1:3	3:2
3	1.5:1	4.0	1:4	2:1

**Table 4:** Orthogonal test schedule.

Sample (#)	Factor A Core–Wall Ratio	Factor B Emulsifier Concentration (%)	Factor C Mass Ratio of Tween-80 to SDBS	Factor D Oil–Water Ratio
1	1:1	2.0	1:2	1:1
2	1:1	3.0	1:3	3:2
3	1:1	4.0	1:4	2:1
4	1.2:1	2.0	1:3	2:1
5	1.2:1	3.0	1:4	1:1
6	1.2:1	4.0	1:2	3:2
7	1.5:1	2.0	1:4	3:2
8	1.5:1	3.0	1:2	2:1
9	1.5:1	4.0	1:3	1:1

**Table 5:** Material list for orthogonal experiment.

Sample (#)	CS (g)	Acetic Acid (mL)	Deionized Water for Acetic Acid (mL)	Tween-80 (g)	SDBS (g)	Deionized Water for Emulsifier (mL)	TTO (g)	STPP (g)
1	1.000	0.990	98.010	0.667	1.333	98.000	1.000	0.400
2	1.000	0.657	65.010	0.750	2.250	97.000	1.000	0.400
3	1.000	0.490	48.510	1.000	3.000	96.000	1.000	0.400
4	1.000	0.490	48.510	0.500	1.500	98.000	1.200	0.400
5	1.000	0.990	98.010	0.600	2.400	97.000	1.200	0.400
6	1.000	0.657	65.010	1.333	2.667	96.000	1.200	0.400
7	1.000	0.657	65.010	0.400	1.600	98.000	1.500	0.400
8	1.000	0.490	48.510	1.000	2.000	97.000	1.500	0.400
9	1.000	0.990	98.010	1.000	3.000	96.000	1.500	0.400

**Table 6:** Materials for single-factor test.

Sample (#)	CS (g)	Acetic Acid (mL)	Deionized Water for Acetic Acid (mL)	Tween-80 (g)	SDBS (g)	Deionized Water for Emulsifier (mL)	TTO (g)	STPP (g)
10	2.000	0.100	99.000	0.400	1.600	198.000	2.400	0.400
11	2.000	0.100	99.000	0.800	3.2000	196.000	2.400	0.400
12	2.000	0.100	99.000	1.200	4.800	194.000	2.400	0.400
13	2.000	0.100	99.000	1.600	6.400	192.000	2.400	0.400
14	2.000	0.100	99.000	2.000	8.000	190.000	2.400	0.400
15	2.000	0.100	99.000	2.400	9.600	188.000	2.400	0.400

**Table 7:** Analysis of microcapsule yield results.

Sample (#)	Factor A Core–Wall Ratio	Factor B Emulsifier Concentration (%)	Factor C Mass Ratio of Tween-80 to SDBS	Factor D Oil–Water Ratio	Output (g)	Yield (%)
1	1:1	2.0	1:2	1:1	1.10	20.37
2	1:1	3.0	1:3	3:2	2.03	33.44
3	1:1	4.0	1:4	2:1	2.33	33.77
4	1.2:1	2.0	1:3	2:1	1.17	22.94
5	1.2:1	3.0	1:4	1:1	1.64	24.85
6	1.2:1	4.0	1:2	3:2	3.12	42.92
7	1.5:1	2.0	1:4	3:2	0.82	14.72
8	1.5:1	3.0	1:2	2:1	1.85	28.91
9	1.5:1	4.0	1:3	1:1	3.20	40.51
Mean value 1	29.193	19.343	30.733	28.577		
Mean value 2	30.237	29.067	32.297	30.360		
Mean value 3	28.047	39.067	24.447	28.540		
Range	2.19	19.723	7.85	1.82		
Factor primary and secondary level	B > C > A > D					
Optimal level	A2	B3	C2	D2		
Optimal scheme	A2 B3 C2 D2					

**Table 8:** Variance analysis table of yield rate.

Factors	Deviation Sum of Squares	Free Degree	F-Ratio	F-Critical Value	Significance
A	7.199	2	0.041	4.460	
B	583.553	2	3.331	4.460	
C	103.589	2	0.591	4.460	
D	6.494	2	0.037	4.460	
Error	700.84	8			

**Table 9:** Analysis of microcapsule coverage rate results.

Sample (#)	Factor A Core–Wall Ratio	Factor B Emulsifier Concentration (%)	Factor C Mass Ratio of Tween-80 to SDBS	Factor D Oil–Water Ratio	Coverage Rate (%)
1	1:1	2.0	1:2	1:1	62.5
2	1:1	3.0	1:3	3:2	57.5
3	1:1	4.0	1:4	2:1	60.0
4	1.2:1	2.0	1:3	2:1	67.5
5	1.2:1	3.0	1:4	1:1	57.5
6	1.2:1	4.0	1:2	3:2	50.0
7	1.5:1	2.0	1:4	3:2	57.5
8	1.5:1	3.0	1:2	2:1	47.5
9	1.5:1	4.0	1:3	1:1	45.0
Mean value 1	60.000	62.500	53.333	55.000	
Mean value 2	58.333	54.167	56.667	55.000	
Mean value 3	50.000	51.667	58.333	58.333	
Range	10.000	10.833	5.000	3.333	
Factor primary and secondary level	B > A > C > D				
Optimal level	A1	B1	C3	D3	
Optimal scheme	A1 B1 C3 D3				

**Table 10:** Variance analysis table of coverage rate.

Factors	Deviation Sum of Squares	Free Degree	F-Ratio	F-Critical Value	Significance
A	172.222	2	1.616	4.460	
B	193.066	2	1.811	4.460	
C	38.889	2	0.365	4.460	
D	22.222	2	0.208	4.460	
Error	426.39	8			

**Table 11:** Yield rate and coverage rate in single factor test.

Sample (#)	Emulsifier Concentration (%)	Yield (%)	Coverage Rate (%)
10	1.0	24.49	45.0
11	2.0	26.84	47.5
12	3.0	31.86	50.0
13	4.0	32.10	57.5
14	5.0	35.19	47.5
15	6.0	30.56	50.0

**Table 12:** The actual number of recovered viable colonies and antimicrobial rate of two types of bacteria on the coating surface.

Sample (#)	Average Number of Recovered Bacteria (CFU/piece)		Antimicrobial Rate (%)	
	*Escherichia coli*	*Staphylococcus aureus*	*Escherichia coli*	*Staphylococcus aureus*
0	207	186	-	-
10	80	108	61.35	41.64
11	155	83	25.12	55.38
12	109	97	47.34	47.85
13	56	45	72.95	75.81
14	64	85	70.53	54.30
15	81	47	60.87	74.73

**Table 13:** The influence of emulsifier concentration on the chromaticity value and color difference of coatings.

Sample (#)	*L*	*a*	*b*	*ΔE*
0	81.43	1.60	1.60	-
10	82.20	1.67	2.60	1.26
11	81.63	0.97	1.37	0.70
12	81.73	1.43	1.47	0.37
13	84.10	1.57	0.83	2.77
14	81.83	1.93	2.10	0.72
15	83.37	2.10	1.27	2.02

**Table 14:** Effect of emulsifier concentration on color difference and light loss rate of coating.

Sample (#)	Glossiness (GU)			Light Loss Rate (%)		
	20°	60°	85°	20°	60°	85°
0	16.2	29.3	37.1	-	-	-
10	15.3	27.9	32.8	5.56	4.78	11.59
11	14.3	24.3	31.2	11.73	17.06	15.90
12	15.7	26.7	33.3	3.09	8.87	10.24
13	11.3	22.8	28.3	30.35	22.18	23.72
14	10.3	23.3	29.5	36.42	20.48	20.49
15	11.5	22.7	24.8	29.01	22.53	33.15

**Table 15:** The influence of emulsifier concentration on the transmission of coatings.

Sample (#)	Transmittance (%)
0	95.65
10	86.34
11	84.67
12	88.42
13	87.80
14	86.24
15	86.28

**Table 16:** The influence of emulsifier concentration on coating roughness.

Sample (#)	Roughness (μm)
0	0.267
10	0.281
11	0.269
12	0.423
13	0.304
14	0.322
15	0.344

**Table 17:** The influence of emulsifier concentration on coating elongation.

Sample (#)	Elongation (%)	*σ* (MPa)	*E* (GPa)
0	20.73	4.84	0.23
10	7.63	4.37	0.57
11	15.17	4.53	0.30
12	16.09	4.59	0.29
13	17.47	2.50	0.14
14	16.15	1.64	0.10
15	4.80	2.94	0.61

## Data Availability

Data are contained within the article.
